# Comparative impact of white and black garlic on intestinal homeostasis: barrier protection, inflammation and microbiota modulation

**DOI:** 10.1016/j.crfs.2026.101457

**Published:** 2026-05-25

**Authors:** Joel Girón-Hernández, Sofia Mares-Bou, Andrea Martelli, Piergiorgio Gentile

**Affiliations:** a Department of Applied Sciences, Faculty of Health and Life Sciences, Northumbria University, Newcastle Upon Tyne, NE1 8ST, United Kingdom; b Center for Biomaterials and Tissue Engineering (CBIT), Universitat Politècnica de València, Valencia, 46022, Spain; c Dipartimento di Ingegneria Enzo Ferrari, Università Degli Studi di Modena e Reggio Emilia, Modena, 41125, Italy; d Biomedical Research Networking Centre on Bioengineering, Biomaterials and Nanomedicine (CIBER-BBN), Carlos III Health Institute, Valencia, 46022, Spain

**Keywords:** Thermal aging, Antioxidants, Caco-2/HT29 co-culture, Tight junctions, Untargeted metabolomics, Probiotic compatibility

## Abstract

Thermal aging transforms white garlic (WG) into black garlic (BG), potentially modifying its biological activity; however, its effects on intestinal barrier function and host-microbiota interactions remain insufficiently characterized. This study compared WG and BG extracts in terms of composition, antioxidant capacity, antimicrobial activity, cytocompatibility and modulation of intestinal homeostasis in a lipopolysaccharide (LPS)-challenged Caco-2/HT29 co-culture *in vitro* model. On a dry basis, aging decreased reducing sugars (∼39 to ∼33 g/100g), while markedly increasing antioxidant content and capacity, including total phenolics (∼111 to ∼433 mg gallic acid equivalents/g), proanthocyanidins (∼689 to ∼1423 mg catechin equivalents/g) and ferric reducing antioxidant power (∼23 to ∼136 mmol Fe^2+^/g). Untargeted metabolomics revealed a compositional shift from amino acid- and sulfur-enriched metabolites in WG toward organic acids and nitrogen-containing compounds in BG. BG extract exerted minimal inhibitory effects against probiotic strains (*Escherichia coli* Nissle, 1917 and *Lacticaseibacillus paracasei*) and exhibited greater cytocompatibility (>80% at 1 mg/mL WG; >100% at 10 mg/mL BG). Both extracts significantly reduced LPS-induced interleukin-6 production (84 ± 5% for BG; 89 ± 24% for WG). Notably, BG more effectively preserved epithelial barrier integrity by maintaining transepithelial electrical resistance (TEER), reducing paracellular permeability and preserving zonula occludens-1 (ZO-1) organization. These findings support BG as a functional ingredient for mitigating inflammation-associated intestinal barrier dysfunction.

## Highlights

• Thermal aging reshaped garlic metabolism and redox profile
• Black garlic showed higher phenolics and electron-transfer antioxidant power
• Aging depleted sulfur precursors and amino acids while generating Maillard products
• Black garlic exerted low antimicrobial pressure on probiotic bacteria
• Black garlic preserved epithelial barrier and reduced cytokine release *in vitro*

## Graphical abstract

**Figure:**
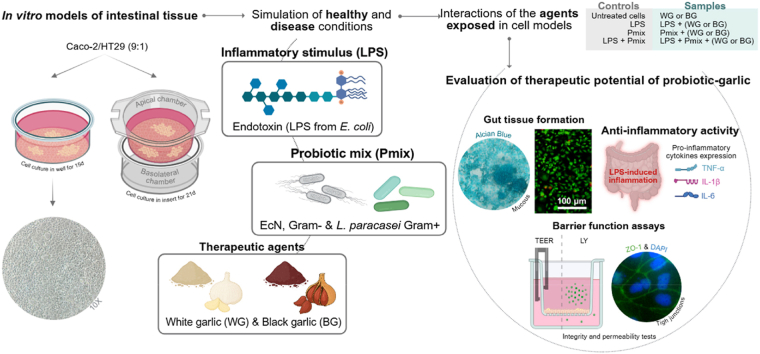

## 1 Introduction

Garlic (*Allium sativum* L., Amaryllidaceae) is one of the oldest known culinary spices and medicinal plants. Traditionally used as flavoring agent, it has gained a reputation for its therapeutic properties to prevent and treat several pathologies, including microbial infections, allergy, hypertension, hypercholesterolemia, diabetes, atherosclerosis and cancer (Banerjee et al., 2002; Manoj Kumar et al., 2017; Tiwari et al., 2009). These biological effects are mainly attributed to its rich content of organosulfur compounds, particularly alliin and its derivatives (e.g., allicin and ajoene), along with other bioactive molecules such as phenolics and flavonoids (Sasmaz et al., 2025; Singh et al., 2025). In addition, several studies have shown that garlic exhibits anti-inflammatory activity by modulating key inflammatory mediators like TNF-α, IL-6 and IL-1β through inhibition of the NF-κB signaling pathway (Hernández‐Cruz et al., 2022). Interest in garlic has expanded beyond its traditional raw form to include value-added products with enhanced functional properties. Among these, black garlic (BG), a traditional food product in parts of East and Southeast Asia, has gained significant scientific and commercial attention due to its improved stability, reduced pungency and superior antioxidant capacity compared to fresh raw garlic (Afzaal et al., 2021; Choi et al., 2014). This transformation is achieved through a controlled thermal aging process that modifies the chemical composition of garlic, resulting in the formation of new compounds such as S-allyl-cysteine (SAC) and melanoidins, which contribute to its improved biological activity (Bae et al., 2014; Liang et al., 2015). Emerging evidence indicates that BG also exerts anti-inflammatory effects by downregulating pro-inflammatory cytokines and suppressing oxidative stress, which are closely linked to chronic inflammation and metabolic disorders (Vinayagam et al., 2023; Sun et al., 2018). As a result of a heat-aging process, garlic undergoes significant changes in flavor, texture and color, largely due to enzymatic and non-enzymatic browning reactions like Maillard reaction, caramelization and oxidation of phenolic compounds. Consequently, the final product acquires a darker color, a softer jelly-like texture and a sweet-balsamic flavor, together with an increased content of bioactive compounds and antioxidant properties compared to raw garlic. These enhanced functional attributes have positioned BG as a promising ingredient in functional foods and nutraceutical applications (Vinayagam et al., 2023; Vecchi et al., 2025; Qiu et al., 2020).

Despite growing evidence that dietary bioactives can modulate intestinal function, the comparative effect of white (raw, WG) and aged BG on intestinal barrier integrity, inflammatory responses and host-microbe interactions remain largely unexplored. Disruption of epithelial tight junctions and chronic low-grade inflammation are central mechanisms underlying intestinal disorders such as inflammatory bowel disease, metabolic endotoxemia and dysbiosis-associated pathologies (Liu, 2024; Colín-González et al., 2017). Therefore, elucidating the functional effects of garlic-derived compounds on the gut epithelium is of considerable translational relevance. In this study we investigated the differential effects of WG and BG extracts on intestinal homeostasis by evaluating: (i) their ability to modulate lipopolysaccharide (LPS)-induced inflammation in Caco-2 and HT29 epithelial cells, (ii) their impact on epithelial barrier permeability, and (iii) their interaction with selected probiotic bacteria (*E. coli* Nissle, 1917 and *L. paracasei*) associated with gut health. We hypothesized that BG, enriched in S-allyl-cysteine and Maillard reaction antioxidants, would exert stronger anti-inflammatory and barrier-protective effects compared with WG, and that both extracts would influence host-microbiota crosstalk under inflammatory conditions.

## 2 Material and methods

### 2.1 Materials and chemicals

Petroleum ether (ACS grade), sulfuric acid (ACS grade), TT-35 Kjeldahl tablets, sodium hydroxide, boric acid with Sher indicator (pH 4.65), hydrochloric acid (ACS grade), Luff-Schoorl reagent, Honeywell Fluka™ absolute ethanol (ACS grade), methanol (HPLC grade), LC/MS-grade H_2_O and acetonitrile (LC-MS grade, 99.8%) were purchased from Fisher Scientific, UK. Potassium iodide (ACS grade, ≥99.0%), sodium thiosulfate (99%), sodium carbonate (ACS grade, ≥99.5%), Folin-Ciocalteu's phenol reagent, D-(+)-glucose (ACS grade), Trolox, deuterium oxide (D_2_O, 99.9% NMR grade, MagniSolv™), Mueller-Hinton broth (MHB), phenol red-free DMEM (DMEM-pr), Triton X-100, phosphate buffered saline 10X (PBS), lipopolysaccharides (LPS) from *E. coli*, bovine serum albumin (BSA), Alcian Blue 8GX and Fluoroshield mounting medium with DAPI, were purchased from Merck, Spain. Gallic acid monohydrate, quercetin (anhydrous), (+)-catechin, and L-ascorbic acid were purchased from Apollo Scientific, UK. Brain heart infusion (BHI) broth (NCM0016B) and BHI agar (NCM0080A) were supplied by Neogen, UK. Human IL-1β, IL-6 and TNF-α ELISA kits were sourced from Abcam, Spain. ReadyProbe™ Cell Viability Imaging Kit, PrestoBlue™ assay, Lucifer Yellow (LY), primary Zona-Occludens 1 (ZO-1) polyclonal antibody and Goat anti-Rabbit IgG (H + L) Cross-Adsorbed Secondary Antibody Alexa Fluor™ 488 conjugated, were supplied by Thermo Fisher Scientific, Spain. All reagents were stored and prepared in accordance with manufacturer guidelines. Distilled water (dH_2_O) was obtained using a Direct-Q 3 water purification system (Merck Millipore, Germany).

### 2.2 White and black garlic preparation

Class I white garlic (*Allium sativum*) harvested in 2024 in China was purchased from a local UK market and processed into black garlic (aged) by thermal treatment. Whole bulbs were sealed in bake-bags and incubated in a circulating air oven (MDE523D, Bartscher, Germany) at 70 °C for 30 days. For functional comparison, cloves from both treatments were freeze-dried for 48 h (Modulyo, Edwards, UK) and ground to powder (A11 basic mill, IKA, Germany) for further analyses. The proximate composition of the samples (n ≥ 3) is provided in the Supplementary Material (S1.1).

### 2.3 Antioxidant capacity and metabolomic profile of garlic samples

Total phenolics (TP) were determined using a modified Folin-Ciocalteu method (Núñez-Ramírez et al., 2025). Total flavonoids (TF) and oligomeric proanthocyanidins (TPA) were analyzed using commercial assay kits (A319717 and A319718, Antibodies, UK). Antioxidant capacity was assessed by ORAC (ab233473, Abcam, USA) and FRAP (Thermo Fisher Scientific, UK) assays, following manufacturer protocols. All measurements were conducted in triplicate and quantified against respective calibration curves. Full methodological details are provided in the Supplementary Material (S1.2).

Garlic powders (10 mg) were extracted with 1 mL methanol, sonicated (15 min, ice-water bath), and centrifuged (15,000 rpm, 4 °C, 15 min) following a previously reported protocol (Girón-Hernández et al., 2024). Supernatants were vacuum-dried (45 °C, 2 h), reconstituted in 100 μL LC/MS-grade water/acetonitrile (95:5), sonicated, filtered and transferred to vials. Extraction blanks and pooled QCs were used for AcquireX MS/MS inclusion/exclusion lists and stability monitoring. LC separation used a Waters HSS T3 column with a water/acetonitrile gradient (A: 95/5, B: 5/95). Data were acquired with Thermo AcquireX and processed in MS-DIAL 4.9, reporting formula, RT, m/z, adduct, MS/MS score, and InChIKey. Full analytical details are provided in the Supplementary Material (S1.3).

### 2.4 Molecular characterization by FTIR-ATR and NMR spectroscopy of white and black garlic

FTIR spectra of freeze-dried samples were recorded using a Frontier FTIR spectrometer (PerkinElmer, UK) with a diamond ATR accessory. Samples were analyzed in triplicate over 4000-550 cm^−1^ (32 scans, 2 cm^−1^ resolution), minimizing atmospheric moisture exposure. ^1^H NMR spectra of white and black freeze-dried garlic were obtained in the liquid state from saturated D_2_O solutions containing TMSP-d_4_ as an internal reference (0.0 ppm). Measurements were acquired at 26.85 °C on an Avance III HD 700 MHz spectrometer (Bruker, UK; BBO probe) using 128 scans (32 K data points, processed to 128 K).

### 2.5 Probiotic sensitivity of white and black garlic

A sensitivity assay was performed to assess the antimicrobial activity of WG and BG extracts against *E. coli* Nissle 1917 (Mutaflor®, Ardeypharm, Germany) and *L. paracasei* subsp. *tolerans* (NCIMB 8822, UK) following a previously reported broth microdilution method (Gallo et al., 2024). Bacterial cultures were activated in brain heart infusion broth (37 °C, ∼20 h), and colony forming units (CFU) were enumerated. Freeze-dried garlic powders were dissolved in Mueller-Hinton broth at up to 100 mg/mL, serially diluted (Table S1), and inoculated with 15 μL bacterial suspension (10^−3^ dilution). After 24 h at 37 °C, growth inhibition was visually assessed. Clear wells were cultured to determine minimum inhibitory (MIC) and bactericidal (MBC) concentrations. Full experimental details are provided in the Supplementary Material (S1.4). All measurements were conducted in triplicate.

### 2.6 Effect of garlic samples on epithelial tissue cytocompatibility and metabolism

Caco-2 and HT29 human colon carcinoma cell lines (Cytion, Germany) were cultured as reported in the Supplementary Material (S1.5). For testing the garlic extracts cytocompatibility, Caco-2 cells were seeded at 20,000 cells/cm^2^ in 48-well plates and cultured for 48 h. Then, garlic samples were tested at concentrations of 50, 10, 5, 1, and 0.5 mg/mL in triplicate. Cell viability was assessed using Live/Dead staining, metabolic activity and cell morphology by immunostaining. Full experimental details are reported in the Supplementary Material (S1.5.1).

### 2.7 Generation of *in vitro* gut-like tissue models under simulated healthy and disease conditions

A Caco-2/HT29 co-culture was established by seeding cells at a 9:1 ratio (33,000 cells/cm^2^) in 48-well plates to model a mixed population of absorptive enterocytes and mucus-secreting goblet cells, mimicking the human intestinal epithelium. Co-cultures were maintained for 21 days and analyzed for epithelial differentiation and barrier organization. Pro-inflammatory cytokines were assessed after stimulating Caco-2/HT29 co-cultures with 10 μg/mL E*. coli* LPS up to 72 h to induce inflammation (Kim et al., 2016). Finally, after removing the LPS-conditioned medium from the apical side of the inserts, a probiotic mixture containing *E. coli* Nissle 1917 (EcN; Gram-, 4.5 × 10^8^ CFU/mL) and *L. paracasei* (Gram+, 6 × 10^7^ CFU/mL) was added, while the basolateral chamber was refreshed with new medium. The co-culture was incubated for 48 h to allow probiotic-cell interactions.

Mucin production was evaluated by Alcian Blue staining. Barrier integrity was assessed by establishing Caco-2/HT29 co-cultures on the apical side of ThinCert® inserts (Greiner Bio-One, Austria) at the same seeding density and culturing them for 21 days. Transepithelial electrical resistance (TEER) was measured throughout this period. To simulate an inflammatory condition, LPS (10 μg/mL) was added to the apical compartment and maintained for up to 48 h. TEER measurements were recorded without medium replacement. Furthermore, paracellular permeability to Lucifer Yellow (LY) was measured to assess tight junction integrity by calculating paracellular permeability (%) as reported by Cummins et al. (2025) with values < 2% indicating intact monolayers and >2% suggesting compromised barrier function. Immunofluorescence analysis of the tight junction protein ZO-1 was assessed with in parallel the determination of cytokine expression in Caco-2/HT29 cultures grown on regular well plates. Full experimental details are reported in the Supplementary Material (S1.5.2).

### 2.8 Effect of white and black garlic on the *in vitro* intestinal-like tissue models

Digested white garlic or black garlic preparations (10 mg/mL, concentration selected from cytocompatibility results) were added and incubated for an additional 48 h. Garlic digestion followed Benlloch-Tinoco et al. (2025), involving sequential incubation in simulated salivary (pH 7, α-amylase 75 U/mL, 2 min), gastric (pH 3, pepsin 2000 U/mL, 2 h) and intestinal (pH 7, pancreatin 100 U/mL, 2 h) fluids at 37 °C and 100 rpm. The digested material was centrifuged, resuspended in PBS or DMEM, and adjusted to the desired concentration. TEER, LY and cytokine expression measurements, and ZO-1 immunostaining were performed.

### 2.9 Statistical analysis

Statistical analyses were performed using GraphPad Prism v9.0. All data (n ≥ 3) are presented as the mean ± SD. Statistical difference was analyzed by one-way analysis of variance (ANOVA), followed by Tukey's multiple comparison test. Significance levels were set at p < 0.05 (∗), p < 0.01 (∗∗) and p < 0.001 (∗∗∗).

## 3 Results

### 3.1 Proximate composition and antioxidant capacity of white and black garlic

**Fig. 1:**
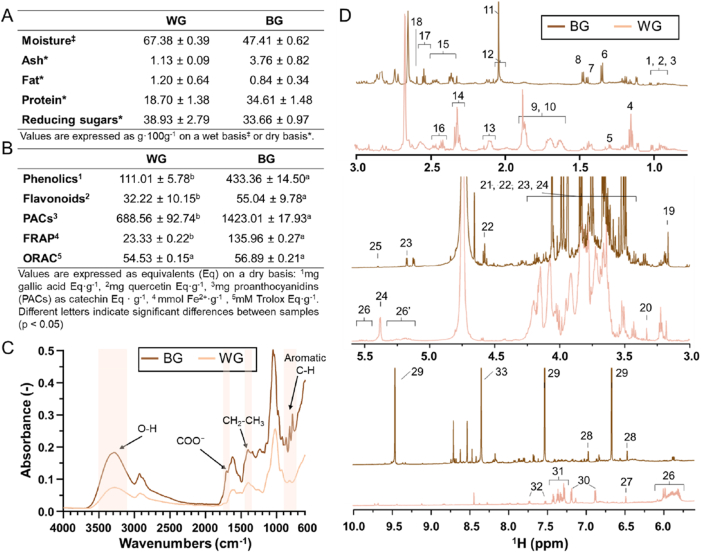
(**A**) Proximate composition of white garlic (WG) and black garlic (BG), moisture is expressed on a wet basis, while ash, fat, protein and reducing sugars are expressed on a dry basis. (**B**) Total phenolics, flavonoids, proanthocyanidins and antioxidant capacity determined by ferric reducing antioxidant power (FRAP) and oxygen radical absorbance capacity (ORAC) of WG and BG expressed on a dry basis. Different superscript letters indicate significant differences between WG and BG (p < 0.05). (**C**) Fourier-transform infrared spectroscopy (FTIR) spectra of WG and BG in the range 4000-600 cm^−1^, highlighting major functional groups and characteristic fingerprint-region signals. (**D**) ^1^H nuclear magnetic resonance (^1^H NMR) spectra of WG and BG extracts showing compositional differences across the aliphatic (0.5-3.0 ppm), sugar and polyol (3.0-5.5 ppm) and aromatic/aldehyde (6.0-10.0 ppm) regions with the following signal assignments:1, valine; 2, leucine; 3, isoleucine; 4, ethanol; 5, threonine; 6, lactic acid; 7, alanine; 8, cycloalliin; 9, lysine; 10, arginine; 11, acetic acid; 12, proline; 13, glutamine; 14, γ-aminobutyric acid (GABA); 15, pyroglutamic acid; 16, glutamic acid; 17, 3-hydroxypropionic acid; 18, succinic acid; 19, choline; 20, methanol; 21, fructose; 22, β-glucose; 23,α-glucose; 24, sucrose; 25, glucosamine; 26, 26′, allicin and other allyl organosulfur compounds; 27, fumaric acid; 28, 5-(hydroxymethyl)-2-furoic acid; 29, 5-(hydroxymethyl)furfural; 30, tyrosine; 31, phenylalanine; 32, tryptophan; 33, formic acid.

The proximate composition of the samples is modified by heat treatment, where the transition from WG to BG involves a series of complex biochemical reactions, resulting in significant compositional changes due to the controlled aging process (Fig. 1A). WG showed higher moisture (67.38 ± 0.39 g/100 g) than BG (47.41 ± 0.62 g/100 g). On a dry basis, BG presented higher protein (34.61 ± 1.48 g/100 g) than WG (18.70 ± 1.38 g/100 g), together with lower reducing sugars (33.66 ± 0.97 vs 38.93 ± 2.79 g/100 g for BG and WG, respectively). Furthermore, for the phenolic content (Fig. 1B), BG exhibited significantly higher total phenolics (433.36 ± 14.50 mg gallic acid equivalents (Eq)/g) than WG (111.01 ± 5.78 mg gallic acid Eq/g). Similarly, flavonoid content was higher in BG (55.04 ± 9.78 mg quercetin Eq/g) compared with WG (32.22 ± 10.15 mg quercetin Eq/g). Proanthocyanidins showed the highest values among the measured bioactive compounds, with BG reaching 1423.01 ± 17.93 mg catechin Eq/g, significantly exceeding WG (688.56 ± 92.74 mg catechin Eq/g). Regarding antioxidant capacity, FRAP values were substantially higher in BG (135.96 ± 0.27 mmol Fe^2+^/g) than in WG (23.33 ± 0.22 mmol Fe^2+^/g). ORAC values were similar between samples, with WG and BG showing 54.53 ± 0.15 and 56.89 ± 0.21 mM Trolox Eq/g, respectively, and no significant differences between them (p < 0.05).

### 3.2 Evaluation of the chemical composition of white and black garlic

FTIR spectra (Fig. 1C) showed a broad band around 3300 cm^−1^, attributed to O-H stretching vibrations of polyhydroxy compounds and water (Kang, 2016). This band was present in both samples but more intense in BG, consistent with higher hydrogen bonding/moisture retention. A peak near 2900 cm^−1^ (asymmetric C-H stretching of methylene groups) was observed in both samples and is typically related to lipids and sugars (Nagarajan et al., 2017). In the fingerprint region (<1500 cm^−1^), BG exhibited stronger absorption at ∼1425 cm^−1^ (CH_2_-CH_3_ bending) and ∼1400 cm^−1^ (O-H bending of carboxylic acids) consistent with the presence of tannins, flavonoids, glycosides and saponins (Choupdar et al., 2024; Alide et al., 2020). Peaks at ∼1030 cm^−1^ in WG and 1020 cm^−1^ in BG were assigned to S=O symmetric stretching vibrations, associated with organosulfur compounds such as allicin and diallyl disulfide. Additional bands in the 900-700 cm^−1^ range were observed in BG, corresponding to aromatic C-H out-of-plane bending and possibly N-H bending of protein residues (Choupdar et al., 2024). ^1^H NMR spectra (Fig. 1D) displayed differences across aliphatic (0.5-3.0 ppm), sugar and polyol (3.0-5.5 ppm) and aromatic/aldehyde (6.0-10.0 ppm) regions. WG showed intense amino acids signals (Liang et al., 2015; Ritota et al., 2012), which were reduced or absent in BG, indicating degradation or conversion during thermal processing (Liang et al., 2015). BG showed increased signals of low-molecular-weight organic acids (lactic, acetic, succinic). In the sugar region, WG was dominated by glucose, fructose and sucrose resonances, whereas BG displayed altered patterns consistent with sugar transformation and non-enzymatic browning (Maillard/caramelization), which generate both flavor-active and antioxidant compounds. In the aromatic region, WG contained signals from aromatic amino acids, while BG exhibited resonances attributable to sugar degradation products (Song et al., 2023).

### 3.3 Metabolomic profile of white and black garlic

**Fig. 2:**
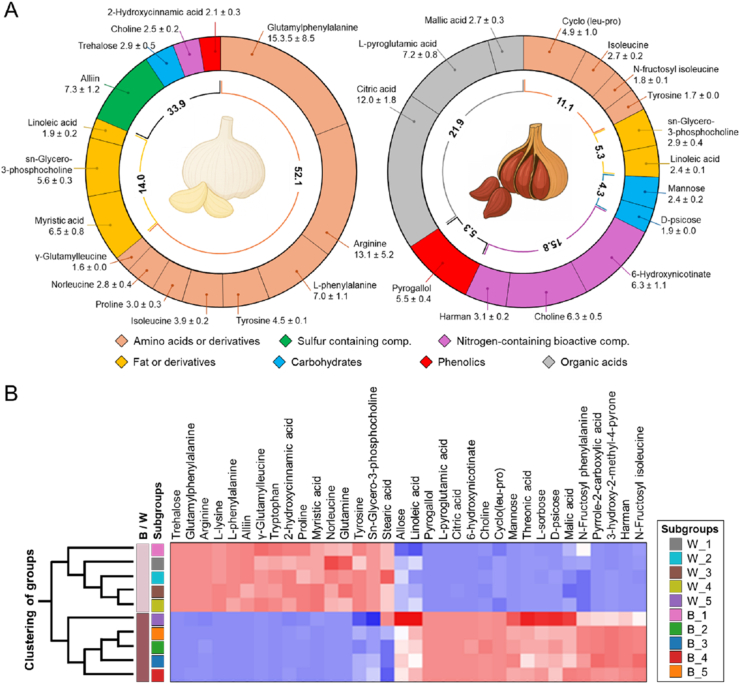
(**A**) Circular plots illustrate the relative contribution (%) of major metabolite classes identified in white garlic (WG, left) and black garlic (BG, right). The inner rings report the proportional abundance of individual metabolite classes, while the outer rings represent subclass distribution. (**B**) The heat map shows the relative abundance of discriminant metabolites between the garlic types; only metabolites exhibiting a statistically significant difference between WG and BG (p < 0.05) were included. Data are displayed as scaled intensities following log_1+x_ transformation and per-metabolite min-max normalization, highlighting relative differences across samples. Red and blue colors indicate higher and lower relative abundance, respectively.

Untargeted metabolomic profiling revealed marked compositional changes following the thermal aging of garlic (Fig. 2A). The top 15 metabolites accounted for 80.0% and 63.9% of the total relative abundance in white and black garlic, respectively. In WG, amino acids and their derivatives predominated (52.1%), followed by fatty acids and related compounds (14.0%). Alliin (7.3%) was the only sulfur-containing metabolite detected in substantial amounts. In contrast, BG showed a profound metabolic remodeling, characterized by the enrichment of organic acids (21.9%) and nitrogen-containing bioactive compounds (15.8%). Amino acids, fatty acids, and sulfur-containing compounds were reduced relative to WG.

Heat map analysis (Fig. 2B), restricted to metabolites differing significantly between WG and BG (p < 0.05), further highlighted distinct metabolomic signatures between the garlic types. Hierarchical clustering of samples produced two main branches corresponding to white (W_1-W_5) and black (B_1-B_5) garlic, showing a consistent separation across biological replicates. The discriminant metabolites formed coherent clusters with opposing abundance patterns: a first cluster displayed higher scaled intensities in WG and lower values in BG, whereas the second cluster showed the reverse pattern, with higher scaled intensities in BG relative to WG. Metabolites contributing to the separation included representatives of amino acid-related compounds and fatty acid-related compounds enriched in WG, and organic acids and nitrogen-containing bioactive compounds more abundant in BG, consistent with the class-level distributions shown in Fig. 2A.

### 3.4 Antimicrobial susceptibility of garlic extracts

**Fig. 3:**
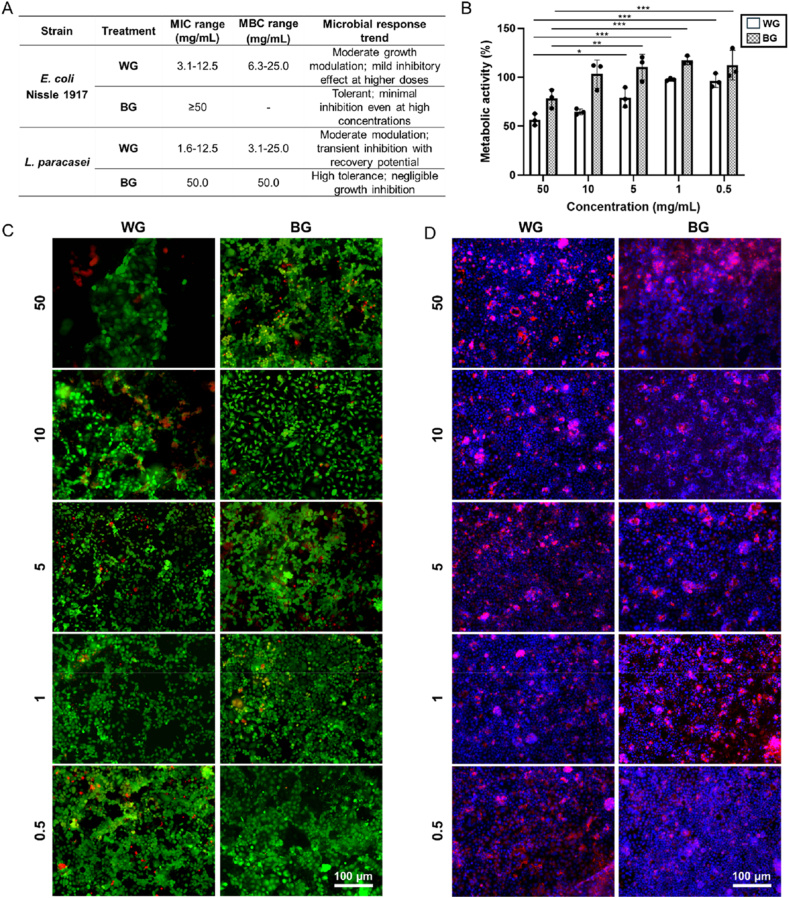
(**A**) Minimum inhibitory concentration (MIC) and minimum bactericidal concentration (MBC) of white garlic (WG) and black garlic (BG) extract. MIC indicates the concentration range of garlic extract that limits bacterial growth. MBC indicates the concentration range at which no viable bacterial cells were detected. Results were obtained from three independent experiments. (**B**) Caco-2 cell metabolic activity determined using the PrestoBlue™ assay following exposure to WG extract or BG extract across the indicated concentration range (50, 10, 5, 1 and 0.5 mg/mL). Data are expressed as percentage metabolic activity relative to untreated control cells. (**C**) Live/dead fluorescence staining of Caco-2 cells after treatment with WG or BG extract, showing live cells (green) and dead cells (red). (**D**) Fluorescence staining of Caco-2 cells after treatment with WG or BG extract, showing cell nuclei stained with 4′,6-diamidino-2-phenylindole (DAPI, blue) and the actin cytoskeleton stained with rhodamine-phalloidin (red).

The antimicrobial activity was evaluated against *E. coli* Nissle 1917 and *L. paracasei* (Fig. 3A). For *E. coli* Nissle 1917, WG showed inhibitory activity with a MIC range of 3.1-12.5 mg/mL and MBC range of 6.3-25.0 mg/mL. The microbial response was characterized by moderate growth modulation, with a mild inhibitory effect observed at higher WG concentrations. In contrast, BG exhibited high tolerance in *E. coli* Nissle 1917, with MIC values ≥ 50 mg/mL and no detectable MBC within all the tested concentration range. For *L. paracasei*, WG displayed inhibitory effects with MIC values ranging from 1.6 to 12.5 mg/mL and MBC values between 3.1 and 25.0 mg/mL. The growth response showed moderate modulation, with transient inhibition followed by recovery potential. BG again showed high tolerance, with both MIC and MBC values at 50.0 mg/mL, and negligible inhibition of *L. paracasei* growth across the tested concentrations.

### 3.5 Cell cytocompatibility of garlic extracts

The cytocompatibility was assessed by metabolic activity and fluorescence-based imaging across a concentration range of 0.5-50 mg/mL (Fig. 3B–D). As shown in Fig. 3B, metabolic activity was maintained above 50% for all tested concentrations of both extracts. WG-treated cells exhibited metabolic activity values ranging from ∼55% to 100%, depending on concentration. At 50 mg/mL, WG resulted in a metabolic activity of ∼55-60%, which significantly increased at lower concentrations, reaching values close to 95-100% at 1 mg/mL. BG-treated cells showed consistently higher metabolic activity than WG at corresponding concentrations, with values ranging from ∼75% at 50 mg/mL to ∼120-130% at 0.5-1 mg/mL. Statistical analysis indicated significant differences between WG and BG at 10, 5, 1 and 0.5 mg/mL (p < 0.05 to p < 0.001), with BG inducing higher metabolic activity. Live/dead fluorescence staining (Fig. 3C) confirmed high cell viability across all concentrations. For both WG and BG, most cells displayed green fluorescence (live cells), with limited red fluorescence (dead cells). At the highest concentration (50 mg/mL), WG-treated samples showed a modest increase in red-stained cells compared with lower concentrations, whereas BG-treated samples maintained predominantly green fluorescence across all tested doses. Finally, nuclear staining and cell distribution analysis (Fig. 3D) further supported these findings, showing preserved cell density and homogeneous nuclear staining for both extracts. Across the concentration range, BG-treated cells exhibited comparable or higher cell density relative to WG-treated cells, with no evident loss of nuclei integrity. Based on these results, a concentration of 10 mg/mL was selected for both WG and BG in the subsequent assays.

### 3.6 Influence of garlic extracts on *in vitro* gut-like epithelial tissue under pathological conditions

**Fig. 4:**
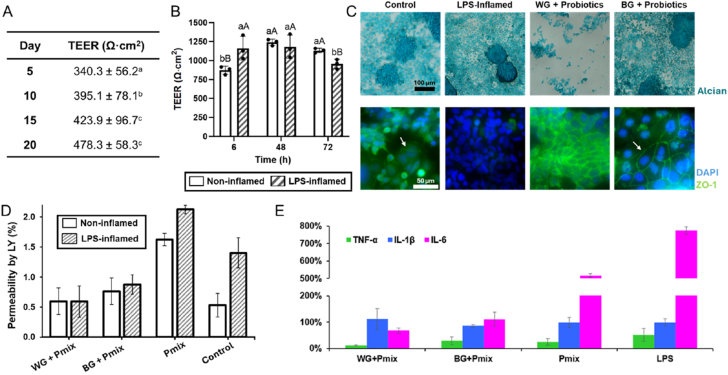
(**A**) Transepithelial electrical resistance (TEER) values measured during the formation and maturation of the gut epithelial tissue cultured on inserts over 5-20 days. (**B**) TEER values of the epithelial model following LPS (lipopolysaccharide)-induced inflammation after 6, 48, and 72 h of treatment. (**C**) Top: Alcian Blue staining of epithelial mucosa after treatment, showing mucin-producing regions and overall tissue morphology. Scale bar = 100 μm; Bottom: Immunofluorescence staining of epithelial monolayers showing tight junction protein zonula occludens-1 (ZO-1, green), with representative junctional structures indicated by arrows, and cell nuclei stained with 4′,6-diamidino-2-phenylindole (DAPI, blue). Scale bar = 50 μm (**D**) Assessment of epithelial impermeability using the Lucifer Yellow (LY) permeability assay. (**E**) Relative levels of inflammatory cytokines tumor necrosis factor alpha (TNF-α), interleukin-1 beta (IL-1β) and interleukin-6 (IL-6) following LPS-induced inflammation, with or without treatment with white garlic (WG) and black garlic (BG). Untreated cells in the absence of the probiotic mixture were used as reference control.

Barrier formation and inflammatory response were evaluated using a Caco-2/HT29 co-culture model to simulate the intestinal epithelium. TEER values increased progressively throughout the 20-day culture period, indicating the establishment of a polarized and functionally integrated epithelial layer (Fig. 4A). Alcian Blue staining confirmed mucus production (Fig. 4C, top), while immunofluorescence analysis showed continuous zonula occludens-1 (ZO-1) localization along cell borders, consistent with the formation of intact tight junctions (Fig. 4C, bottom).

LPS stimulation induced epithelial dysfunction, evidenced by a reduction in TEER after 72 h (Fig. 4B), increased paracellular permeability (Fig. 4D) and levels of IL-1β, IL-6 and TNF-α (Fig. 4E). Treatment with WG or BG extracts attenuated the inflammatory response compared with the LPS group, particularly by reducing IL-6 levels. Among the treatments, BG combined with the probiotic mixture (Pmix) showed the greatest reduction in pro-inflammatory cytokines, whereas Pmix alone only partially mitigated cytokine production and remained substantially higher than the garlic-treated groups. In addition, both WG- and BG-treated cultures exhibited lower LY permeability than the LPS-treated condition, indicating partial preservation of epithelial barrier integrity (Fig. 4D).

## 4 Discussion

Garlic is widely recognized as a functional food with antimicrobial, antioxidant and anti-inflammatory properties, largely attributed to its rich content of organosulfur compounds and polyphenols (Liu, 2024; Arreola et al., 2015). Beyond systemic effects, accumulating experimental evidence indicates that garlic and garlic-derived preparations can modulate intestinal inflammation, enhance epithelial barrier integrity and attenuate colitis-like pathology through suppression of NF-κB-dependent cytokines and preservation of tight junction architecture (Zhan et al., 2022; Vezza et al., 2019). However, despite growing interest in thermally processed garlic products, how aging-induced chemical transformations translate into altered interactions with the gut epithelium and commensal microbiota remains poorly defined (Han et al., 2024). Here, we have demonstrated that thermal aging profoundly remodeled garlic metabolism, generating a microbiota-compatible, barrier-protective and anti-inflammatory phenotype under conditions relevant to gut inflammation. These findings provide new mechanistic insight into how food processing can yield gut-targeted bioactive compounds.

Particularly, thermal processing induced extensive chemical reorganization, shifting fresh WG toward the aged BG profile. This transition involved the conversion of unstable, highly reactive organosulfur compounds into more stable, water-soluble derivatives such as S-allyl-L-cysteine and S-allyl-mercaptocysteine, alongside a marked increase in phenolics and Maillard-derived antioxidant products (Vecchi et al., 2025; Ahmed et al., 2021). These changes are consistent with previous reports describing aging as a key driver of functional diversification in garlic. The increase in phenolic content and antioxidant capacity observed in BG likely arises from multiple, non-mutually exclusive mechanisms, that include thermal cleavage of esterified or glycosylated phenolics, formation of phenolic-related compounds during advanced browning reactions and enhanced extractability resulting from heat-induced disruption of plant cell wall matrices (Sasmaz et al., 2025). In contrast, total flavonoid levels were not substantially affected by aging, in agreement with earlier studies (Kim et al., 2013), suggesting that qualitative rather than quantitative changes dominate the antioxidant shift. Spectroscopic and metabolomic analyses confirmed profound biochemical remodeling. FTIR profiles of BG showed enhanced carbonyl- and carboxylate-associated bands, consistent with accumulation of organic acids, aldehydes and ketones generated through oxidative and Maillard-driven pathways (Liang et al., 2015; Choupdar et al., 2024; Pakakaew et al., 2025). Moreover, NMR profiling further revealed depletion of free amino acids and sulfur-containing precursors, accompanied by increased low-molecular-weight organic acids. Although γ-aminobutyric acid (GABA), a compound with recognized neuroactive and gut-related functions, was reduced following aging, this loss coincided with accumulation of alternative bioactive metabolites, indicating a redistribution rather than depletion of functional potential (Braga et al., 2024). Notably, BG was enriched in nitrogen-containing metabolites such as choline and β-carbolines (e.g., harman). Choline is a biologically active compound involved in membrane integrity, epithelial signaling and immune modulation, and has been linked to host-microbiota interactions in inflammatory settings (Zhan et al., 2023; Huang et al., 2025). Harman, while context-dependent in its biological effects, is best interpreted here as a marker of advanced thermal remodeling and increased redox complexity (Jiménez-Amezcua et al., 2025). In parallel, accumulation of organic acids including citric and malic acid reflects oxidative conversion of primary metabolites during aging (Hu et al., 2024). Such acidification may influence luminal pH and microbial metabolic activity (Oliphant et al., 2019), conditions associated with microbial configurations favoring short-chain fatty acid production, a key contributor to epithelial barrier maintenance and immune homeostasis (Parada Venegas et al., 2019). Sulfur metabolism was also profoundly altered. The disappearance of alliin and related S-alk(en)yl-L-cysteine sulfoxides confirms their thermal instability and accounts for the reduced sulfur reactivity of BG (Parada Venegas et al., 2019). This transition from a sulfur-rich to a nitrogen- and organic acid-enriched profile was reflected in antioxidant behavior, with BG showing a marked increase in electron-transfer-based reducing power compared with WG. This enhancement is plausibly driven by the combined contribution of increased polyphenols, stable sulfur metabolites and Maillard reaction products such as melanoidins (Pakakaew et al., 2022). In contrast, ORAC values differed only marginally between WG and BG, underscoring the assay dependence of antioxidant measurements and suggesting that aging preferentially enhances redox buffering rather than peroxyl radical scavenging (Manoonphol et al., 2023).

Importantly, these metabolomic changes translated into distinct microbiological outcomes. BG exhibited minimal inhibitory effects on probiotic strains, including *E. coli* Nissle 1917 and *L. paracasei*, whereas WG retained stronger growth-modulating capacity, likely due to residual reactive sulfur compounds (Wu et al., 2020). Reduced antimicrobial pressure is a critical feature in the context of intestinal inflammation, where preservation of commensal populations supports epithelial recovery and immune regulation (Booyens et al., 2014). Because commensal preservation and epithelial homeostasis are tightly interdependent in inflamed mucosa, we next examined epithelial responses under inflammatory stress. In LPS-stimulated models, both garlic extracts attenuated cytokine release, but BG exerted significantly stronger suppression of IL-1β, IL-6, and TNF-α. BG, alone or combined with probiotics, maintained cytokine levels close to baseline, indicating robust anti-inflammatory activity without compromising probiotic viability. These effects are consistent with inhibition of NF-κB-dependent signaling by phenolic antioxidants, S-allyl-cysteine and Maillard-derived melanoidins, which collectively dampen oxidative stress and downstream inflammatory cascades (Choi et al., 2014). Crucially, BG preserved epithelial barrier integrity, as shown by maintenance of TEER, reduced paracellular permeability and preserved ZO-1 organization. These findings align with emerging evidence that BG-derived compounds strengthen tight junctions and enhance mucosal resilience (Colín-González et al., 2017). The observed synergy with probiotics further suggests that BG metabolites support, rather than disrupt, commensal-epithelial cross-talk, contrasting with the transient antimicrobial reactivity of WG. Overall, this study demonstrates that thermal aging transforms garlic into a metabolically and functionally distinct food capable of modulating gut inflammation and barrier function. By linking specific metabolic transformations to microbial compatibility, epithelial integrity and cytokine suppression, our findings advance the concept of food-derived immunonutrients targeting intestinal inflammation and barrier dysfunction (Vecchi et al., 2025; Liu, 2024). Black garlic thus emerges as a promising dietary component for restoring epithelial homeostasis and mitigating inflammation-driven intestinal disorders. Nevertheless, some limitations should be acknowledged, as the present study was based exclusively on *in vitro* models that cannot fully reproduce the complexity of the *in vivo* gastrointestinal environment. Therefore, future studies integrating advanced new approach methodologies (NAMs), including multicellular and organ-on-chip platforms, are warranted to better investigate complex intestine-related interactions, such as the gut-brain and gut-immune axes, and to improve the translational relevance of food bioactivity research.

## 5 Conclusions

This study demonstrated that thermal aging transformed garlic into a metabolically and functionally distinct food with enhanced compatibility toward commensal microorganisms and improved anti-inflammatory and barrier-protective effects in intestinal epithelial models. By linking aging-induced metabolic remodeling with modulation of cytokine production, epithelial integrity and probiotic compatibility, our findings supported the potential of black garlic as a promising dietary strategy for promoting intestinal homeostasis and mitigating inflammation-associated gut dysfunction.

## CRediT authorship contribution statement

JGH: Conceptualization, Methodology, Investigation, Formal analysis, Writing - review & editing, Supervision, Project administration, Funding acquisition. SMB: Methodology, Investigation, Formal analysis, Data curation, Writing - original draft. AM: Methodology, Investigation, Formal analysis, Data curation, Writing - original draft. PG: Conceptualization, Methodology, Investigation, Formal analysis, Writing - review & editing, Supervision, Project administration, Funding acquisition.

## Data availability statement

Data supporting this study are openly available from Universitat Politècnica de València's RiuNet https://riunet.upv.es/handle/10251/231527.

## Declaration of generative AI and AI-assisted technologies in the writing process

In the preparation of this work, the author(s) used ChatGPT for assistance with proof-editing. After utilizing this tool/service, the author(s) carefully reviewed and revised the content as needed, assuming full responsibility for the final publication.

## Declaration of competing interest

The authors declare that they have no known competing financial interests or personal relationships that could have appeared to influence the work reported in this paper.
