# Supplementary material for: Comparative impact of white and black garlic on intestinal homeostasis: barrier protection, inflammation and microbiota modulation
Source: Curr Res Food Sci. 2026 May 25;12:101457. doi: 10.1016/j.crfs.2026.101457 (PMC13235496; doi:10.1016/j.crfs.2026.101457)
Supplement: Multimedia component 1 [file mmc1.docx]

**Supplementary Material**

**Comparative impact of white and black garlic on intestinal homeostasis: barrier protection, inflammation, and microbiota modulation**

Joel Girón-Hernández^ab*^, Sofia Mares Bou^b^, Andrea Martelli^c^, Piergiorgio Gentile^bd*^

^a^ Department of Applied Sciences, Faculty of Health and Life Sciences, Northumbria University, NE1 8ST Newcastle Upon Tyne, United Kingdom

^b^ Center for Biomaterials and Tissue Engineering (CBIT), Universitat Politècnica de València, 46022 Valencia, Spain

^c^ Dipartimento di Ingegneria Enzo Ferrari, Università degli Studi di Modena e Reggio Emilia, 41125 Modena, Italy

^d^ Biomedical Research Networking Centre on Bioengineering, Biomaterials and Nanomedicine (CIBER-BBN), Carlos III Health Institute, 46022 Valencia, Spain

**S1. METHODS**

**S1.1 Proximate composition**

*Moisture content.* Moisture content was determined gravimetrically by drying 5 g of homogenized black and white garlic in a UF30 forced-air oven (Memmert, Germany) at 105 °C for 24 h. The dried sample was cooled in a desiccator before reweighing. Moisture content was calculated as the weight loss percentage.

*Ash content.* Both white and black garlic samples (not freeze-dried) were finely chopped/crushed, and 1 g was placed in a pre-weighed crucible. The samples were ashed at 550 °C for 1.5 h in an AAF 1100 furnace (Carbolite, UK). After cooling in a desiccator with silica gel, the final weight was recorded. Ash content was calculated as a percentage of the initial sample weight.

*Fat content.* Fat content was determined gravimetrically using Soxhlet extraction. Dried, ground food samples (black and white) were placed in a pre-weighed cellulose thimble and extracted with petroleum ether (40–60 °C) for 6-8 h. The solvent was then evaporated, and the residual fat was dried at 105 °C, cooled in a desiccator, and weighed. Fat content was calculated as the lipid mass percentage relative to the initial dry sample weight.

*Protein content*. Protein content was determined using the Kjeldahl method. Approximately 1 g of the sample was digested with H_2_SO_4_ and a catalyst, then neutralized with NaOH, releasing ammonia, which was distilled into boric acid. The ammonia was titrated with standardized HCl, and protein content was calculated using the vegetables conversion factor.

*Reducing sugars.* Reducing sugars were determined using the Luff-Schoorl titration method. 25 mL of Luff-Schoorl reagent was mixed with 10 mL of 1:80 (g/mL) either the sample or standard glucose solution 4:6 (vol/vol) ethanol/ dH_2_O and heated in a boiling water bath for 10 min. After rapid cooling, 10 mL of 1 M H_2_SO_4_ was added, followed by 10 mL of 10% KI, releasing iodine. The iodine was titrated with 0.1 M Na_2_S_2_O_3_ until pale yellow, then 2–3 drops of starch indicator were added, and titration continued until the blue color disappeared. Reducing sugar content was calculated by comparing Na_2_S_2_O_3_ volumes between the sample and standard.

**S1.2 Antioxidant capacity, activity and Metabolomic profile of garlic samples**

***Total Phenolic (TP) Determination.* Total phenolic content was determined using a modified Folin-Ciocalteu method. A 50 µL sample (1 mg/mL) was mixed with 430 µL deionized water and 20 µL Folin-Ciocalteu reagent, followed by the addition of 50 µL Na_2_CO_3_ (20 g/100 mL). The mixture was incubated in darkness for 10 min, then diluted with 450 µL dH_2_O. A 200 µL aliquot was transferred to a 96-well plate, and absorbance was measured at 680 nm using a FLUOstar UV-Vis spectrophotometer (BMG Labtech, Germany). Quantification was performed using a gallic acid calibration curve (0.05–1 mg/mL, R^2^ = 0.997), with results expressed as mg gallic acid equivalents (GAE)/g sample.**

*Total Flavonoid (TF) Determination.* Total flavonoid content was analyzed using the A319717-Plant Flavonoids Assay (Antibodies, UK). Samples (1 mg/mL) in 6:4 (vol/vol) ethanol/ dH_2_O solution were sequentially mixed with 15 μL nitrite solution (5 min), 15 μL chromogen (5 min), 120 μL NaOH, and 90 μL 60% ethanol (15 min). Absorbance was recorded at 502 nm using a 96-well plate reader. A quercetin calibration curve (0.0156-1 mg/mL, R^2^ = 0.999) was employed, with results expressed as mg quercetin equivalents (QE)/g sample.

*Total Oligomeric Proanthocyanidins (TPA) Determination.* Total oligomeric proanthocyanidins were quantified using the A319718-Plant Oligomeric Proanthocyanidins Assay Kit (Antibodies, UK). Samples (1 mg/mL in extraction buffer) were combined with 160 μL reagent in a 96-well plate and incubated at 30 °C for 30 min. Absorbance was measured at 500 nm, and concentrations were calculated using a catechin standard curve (0.039–5 mg/mL, R^2^ = 0.996), with results expressed as mg catechin equivalents (CE)/g sample.

*Oxygen Radical Absorbance Capacity (ORAC) Determination.* The ORAC assay was conducted using the ab233473-ORAC Assay Kit (Abcam, USA). Samples (0.02 mg/mL in 1× assay diluent) were mixed with 150 μL fluorescein solution, incubated at 37 °C for 30 min, and fluorescence was recorded (Ex/Em = 480/520 nm). After adding 25 μL Free Radical Initiator (80 mg/mL), fluorescence was continuously monitored every minute for 1 h. A Trolox standard curve (0–50 μM, R^2^ = 0.980) was used, with results expressed as M Trolox equivalents (TE)/g sample.

*Ferric Reducing Antioxidant Power (FRAP) Determination.* FRAP was measured using the ferric antioxidant status detection Kit (Thermo Fisher Scientific, UK). Samples (1 mg/mL, 1:10 dilution) were combined with 75 μL FRAP reagent in a 96-well plate and incubated for 30 min. Absorbance was recorded at 560 nm, and an ascorbic acid standard curve (0.05–1000 μM, R^2^ = 0.999) was used to determine results, expressed as mmol Fe^2+^/g sample.

All analyses were performed at least in triplicate, following manufacturer guidelines.

**S1.3 Metabolomic profile**

Sample powders (10 mg) were extracted in 1 mL of analytical methanol, sonicated for 15 min in an ice-water bath, and centrifuged at 15,000 rpm, 4 °C, for 15 min. Supernatants were vacuum-dried (45 °C, 2 h), resuspended in 100 μL of 95/5 LC/MS-grade water/acetonitrile, sonicated, and filtered (0.22 μm Costar Spin X, 10,000 rpm, 5 min). Filtrates were transferred to autosampler vials with 200 μL micro-inserts. Extraction blanks and pooled QCs were included for MS/MS exclusion lists, inclusion lists, and stability assessment. Separation was performed on a Waters HSS T3 column (2.1 × 150 mm, 1.7 μm, 35 °C) with a 250 μL min^-1^ flow rate using a binary buffer system: Buffer A (95/5 LC/MS-grade water/acetonitrile) and Buffer B (5/95 LC/MS-grade water/acetonitrile). The gradient was: T_0_ 1.5 min, 95% A; T_1.5_ 11.5 min, 5% A; T_15_ 20 min, 95% A, with a 21.5 min injection-to-injection time. Data acquisition followed Thermo AcquireX with MS1 resolution at 30,000 (100-1000 m/z, Quad RF frequency 30, AGC 95%, max injection time 50 ms) and MS2 using stepped HCD (10, 35, 60) with 1.0 m/z isolation width. Instrument parameters: vaporizer temp (275 °C), ion transfer tube temp (300 °C), and gas flow (sheath: 35, aux: 7, sweep: 0). Data processing involved .abf file conversion and alignment using Riken MSDial 4.9. MS2 matches (≥70% similarity, 10-ppm tolerance) were verified against an authentic compound library. QC metrics (RSD ≤1.3%, threshold ≤15%) ensured stable MS/MS signals (RSD ≤25%), with associated metadata: formula, S/N ratio, retention time, m/z, adduct, MS/MS match score, and InChIKey.

**S1.4 Probiotic interaction of white and black garlic**

A sensitivity assay was conducted to evaluate the antimicrobial effects of white and black garlic extracts against *E. coli Nissle* 1917 (Mutaflor®, Ardeypharm, Germany), *L. paracasei*, Lacticaseibacillus paracasei subsp. tolerans (NCIMB 8822, UK). Bacterial cultures were activated by suspending a single colony in ~10 mL of brain heart infusion (BHI) broth and incubating at 37 °C for ~20 h. The initial bacterial load was determined by colony-forming unit (CFU) enumeration. Freeze-dried garlic powder (raw and fermented) was dissolved in 1 mL of Mueller-Hinton broth (MHB) in a multiwell plate. The test volume was set at 150 μL, with a maximum concentration of 100 mg of garlic sample per mL, followed by serial dilutions in MHB (table S1). A 15 μL aliquot of bacterial suspension (10^-3^ dilution) was added to each well. After incubation at 37 °C for ~24 h, bacterial growth was visually assessed. From wells with clear media (indicating inhibition), 10 μL was transferred to 90 μL of BHI, plated onto MHI agar, and incubated at 37 °C for ~24 h. Colony presence determined the minimum bactericidal concentration (MBC), while surviving colonies were counted to establish the minimum inhibitory concentration (MIC).

**Table S1**. Experimental setup for bacterial sensitivity analysis

| **Concentration**  **(mg/ml)** | **Stock garlic 110 (µL)** | **MHB**  **(µL)** | **Bacteria**  **(µL)** | **Set volumen (µL)** |
| --- | --- | --- | --- | --- |
| 100.00 | 138.6 | 1.4 | 10 | 150 |
| 50.00 | 136.4 | 3.6 | 10 | 150 |
| 25.00 | 68.2 | 71.8 | 10 | 150 |
| 12.50 | 34.1 | 105.9 | 10 | 150 |
| 5.00 | 13.6 | 126.4 | 10 | 150 |
| 2.50 | 6.8 | 133.2 | 10 | 150 |
| 1.25 | 3.4 | 136.6 | 10 | 150 |
| 0.50 | 1.4 | 138.6 | 10 | 150 |
| 0.00 | 0.0 | 140.0 | 10 | 150 |

Notes: A stock concentration of 110 mg/ mL of garlic powder was prepared in Mueller-Hinton broth (MHB). Additionally, a well containing 150 μL of MHB without inoculum was included as a control.

**S1.5 Cell tissue *in vitro* evaluation**

Caco-2 and HT29 human colon carcinoma cell lines (Cytion, Germany) were cultured in Dulbecco’s Modified Eagle Medium (DMEM) supplemented with 10% fetal bovine serum and 1% penicillin/streptomycin. Cells were maintained at 37 °C in a humidified 5% CO_2_ atmosphere.

**S1.5.1 Effect of garlic samples on Caco-2/HT29 cytocompatibility and metabolism**

Caco-2 cells were seeded at 20,000 cells/cm^2^ in 48-well plates and cultured for 48 h. Garlic samples were tested at concentrations of 50, 10, 5, 1, and 0.5 mg/mL.

Cell viability was assessed using Live/Dead staining (ReadyProbes™, Thermo Fisher Scientific, UK). After washing with PBS, cells were incubated with 150 μL of a staining solution containing 4 μM ethidium homodimer-1 and 2 μM calcein in PBS for 30 min at room temperature. Fluorescence images were acquired with an EVOS M5000 microscope (Thermo Fisher Scientific, UK) using excitation/emission wavelengths of 488/515 nm (calcein) and 570/602 nm (ethidium homodimer-1).

Metabolic activity was determined using the PrestoBlue™ assay (Thermo Fisher Scientific, UK). Cells were incubated for 1.5 h with a 10% (v/v) reagent, and fluorescence was measured using a FLUOstar Omega plate reader (BMG Labtech, Germany).

Cell morphology was examined after fixation with 4% paraformaldehyde for 15 min, permeabilisation with 0.1% (v/v) Tween 20® in PBS, and staining with rhodamine-phalloidin (1:100, Sigma-Aldrich, P1951) for 30 min. Nuclei were counterstained with DAPI (VECTASHIELD® antifade medium) and imaged with a fluorescence microscope.

**S1.5.2 Generation of intestinal tissue under simulated healthy and disease conditions**

A Caco-2/HT29 co-culture was established by seeding cells at a 9:1 ratio (33,000 cells/cm^2^) in 48-well plates to model a mixed population of absorptive enterocytes and mucus-secreting goblet cells, mimicking the human intestinal epithelium. Co-cultures were maintained for 21 days and analysed for epithelial differentiation and barrier organization. Pro-inflammatory cytokines were assessed after stimulating Caco-2/HT29 co-cultures with 10 µg/mL *E. coli* LPS up to 72 h to induce inflammation.

Mucin production was evaluated by Alcian Blue staining (Sigma-Aldrich, UK). Samples were fixed overnight at 4 °C in 4% paraformaldehyde, rinsed with PBS, and incubated for 5 min with Alcian Blue (1 g/mL in dH_2_O, pH 2.5 adjusted with acetic acid). Excess dye was removed by PBS washing, and images were acquired using an Eclipse 80i optical microscope equipped with a Digital Sight 10 camera (Nikon, Japan).

Barrier integrity was assessed by establishing Caco-2/HT29 co-cultures on the apical side of ThinCert® inserts (0.4 µm pore size; Greiner Bio-One, Austria) at the same seeding density and culturing them for 21 days. Transepithelial electrical resistance (TEER) was measured throughout this period using an EVOM2 ohmmeter equipped with an STX2 electrode on a warming plate maintained at 37 °C (World Precision Instruments, USA). During each measurement, the culture medium in both the apical and basolateral compartments was replaced with phenol red-free DMEM, and regular medium was reintroduced immediately afterward. To simulate an inflammatory condition, LPS (10 µg/mL) was added to the apical compartment and maintained for up to 48 h. TEER measurements were recorded at 6, 48, and 72 h without medium replacement.

**S1.5.3 Effect of probiotics and garlic**

After removing the LPS-conditioned medium from the apical side of the inserts, a probiotic mixture containing *E. coli* Nissle 1917 (EcN; Gram–, 4.5 × 10^8^ CFU/mL) and *L. paracasei* (Gram+, 6 × 10^7^ CFU/mL) was added, while the basolateral chamber was refreshed with new medium. The co-culture was incubated for 48 h to allow probiotic-cell interactions.

Subsequently, digested white garlic or black garlic preparations (10 mg/mL) were added and incubated for an additional 48 h. Garlic digestion involved sequential incubation in simulated salivary (pH 7, α-amylase 75 U/mL, 2 min), gastric (pH 3, pepsin 2000 U/mL, 2 h), and intestinal (pH 7, pancreatin 100 U/mL, 2 h) fluids at 37 °C and 100 rpm. The digested material was centrifuged, resuspended in PBS or DMEM, and adjusted to the desired concentration.

**S1.5.4 Evaluation of the therapeutic potential of probiotic-garlic treatment in intestinal inflammation**

Histological evaluation of treated co-cultures was performed by Alcian Blue staining as described above. Paracellular permeability to Lucifer Yellow (LY, Thermo Fisher Scientific, Spain) was measured to assess tight junction integrity. After treatment, media were removed from both compartments. LY solution (500 µL, 0.1 mg/mL in phenol red-free DMEM) was added to the apical chamber and 1,000 µL of phenol red-free DMEM to the basolateral side. Plates were incubated for 60 min at 37 °C, and 150 µL from the basolateral medium was transferred to a black 96-well plate for fluorescence measurement (485 nm excitation/535 nm emission). Blank and positive controls were included. Paracellular permeability (%) was calculated as described in Eq. 1.

$Paracellular permeability (\%)=\frac{F_{sample}-F_{blank}}{F_{positive control}-F_{blank}}$ (Eq. 1)

where F is the fluorescence signal measured in the basolateral compartment.

For immunofluorescence analysis of the tight junction protein ZO-1, cells were fixed, permeabilised, and blocked as previously described. Samples were incubated overnight at 4 °C with an anti-ZO-1 antibody (1:150, Thermo Fisher Scientific, Spain) in 0.05% Triton X-100, followed by a 1 h incubation at room temperature with an Alexa Fluor 488-conjugated secondary antibody (1:2,000) in 0.05% Triton X-100, protected from light. Nuclei were counterstained with DAPI-containing mounting medium. Images were captured using a Eclipse 80i microscope equipped with a Digital Sight 10 camera (Nikon, Japan) with DAPI (blue nuclei) and FITC (green ZO-1) filters.

Cytokine expression was determined in Caco-2/HT29 cultures grown on regular well plates following 24 h exposure to garlic samples and selected probiotics. Supernatants were collected, centrifuged (4,000 rpm, 5 min, 4 °C), and analysed for IL-1β, IL-6, and TNF-α using ELISA kits (Abcam, USA) according to the manufacturer’s protocol. Absorbance was recorded at 450 nm, and cytokine concentrations were calculated from standard curves (R^2^= 0.99).
